# MicroRNA-26b inhibits epithelial-mesenchymal transition in hepatocellular carcinoma by targeting USP9X

**DOI:** 10.1186/1471-2407-14-393

**Published:** 2014-06-02

**Authors:** Gang Shen, Ye Lin, Xuewei Yang, Jing Zhang, Zhe Xu, Hongyun Jia

**Affiliations:** 1 Department of interventional Radioloby and Vascular Anomalies, Affilated Guangzhou women and children’s medical center of Guangzhou Medical University, Guangzhou, China; 2 Department of general surgery, Guangdong Gerernal Hospital, Guangzhou, China; 3 Department of Hepatobiliary Surgery, the second affiliated hospital of Guangzhou Medical University, Guangzhou, China; 4 Department of Pediatric Surgery, First Affiliated Hospital of Sun Yat-sen University, Guangzhou, China; 5 Department of clinical examination, the second affiliated hospital of Guangzhou Medical University, Guangzhou, China

**Keywords:** miR-26, USP9X, Epithelial-mesenchymal transition, Hepatocellular carcinoma

## Abstract

**Background:**

Metastasis is responsible for the rapid recurrence and poor survival of malignancies. Epithelial-mesenchymal transition (EMT) has a critical role in metastasis. Increasing evidence indicates that EMT can be regulated by microRNAs (miRNAs). The aim of this study was to investigate the role of miR-26b in modulating epithelial-mesenchymal transition (EMT) in hepatocellular carcinoma (HCC), as well as to identify its underlying mechanism of action.

**Methods:**

The expression level of miR-26b was assessed in multiple HCC cell lines (HepG2, MHCC97H, Hep3B, MHCC97L, HCCC9810, BEL-7402, Huh7 and QGY-7703), as well as in liver tissue from patients with HCC. Follow-up studies examined the effects of a miR-26b mimic (increased expression) and a miR-26b inhibitor (decreased expression) on markers of EMT, wound healing and cell migration. The molecular target of miR-26b was also identified using a computer algorithm and confirmed experimentally.

**Results:**

MiR-26b expression was decreased in HCC cell lines and was inversely correlated with the grade of HCC. Increased expression of miR-26b inhibited the migration and invasiveness of HCC cell lines, which was accompanied by decreased expression of the epithelial marker E-cadherin and increased expression of the mesenchymal marker vimentin, at both the mRNA and protein expression levels. A binding site for miR-26b was theoretically identified in the 3′UTR of USP9X. Further studies revealed that overexpression of miR-26b repressed the endogenous level of USP9X protein expression. Overexpression of miR-26b also repressed Smad4 expression, whereas its inhibition elevated Smad4 expression.

**Conclusions:**

Taken together, our results indicate that miR-26b were inhibited in HCC. In HCC cell lines, miR-26b targeted the 3′UTR of USP9X, which in turn affects EMT through Smad4 and the TGF-β signaling pathway. Our analysis of clinical HCC samples verifies that miR-26b also targets USP9X expression to inhibit the EMT of hepatocytes. Thus, miR-26b may have some effects on the EMT of HCC cells.

## Background

Hepatocellular carcinoma (HCC) is a common and aggressive cancer, with an increasing incidence globally, especially in China [1]. Despite technical advances and improved surgical treatment, the rate of tumor recurrence and metastasis after curative resection remains high [2,3]. Exploring the molecular mechanisms underlying the initiation, progression and metastasis of HCC is vital as it may provide new therapeutic targets, leading to improvements in the long-term survival of patients with HCC [4]. Although the genetic events responsible for HCC initiation and progression are not clear, they involve at least three carcinogenic pathways: the p53, NF-κB and transforming growth factor (TGF-β) signaling pathways [5-7].

The TGF-β signaling pathway is particularly pertinent to the current study, as it is known to play a central role in tumorigenesis and tumor progression by regulating many critical cellular processes, including cell proliferation, apoptosis and epithelial-mesenchymal transition (EMT) [7]. Furthermore, TGF-β has been shown to have a central role in the growth of hepatocytes [8]. With regard to the progression of HCC, it has previously been shown in HCC cell lines, such as Hep3B, HepG2, PLC/PRF5, that TGF-β signaling triggers EMT [9], characterized by lower E-cadherin expression and high vimentin expression in vitro [10]. There is also convincing evidence that TGF-β signaling can induce EMT in mouse hepatocytes *in vitro*[11]. Subsequent studies revealed the mechanism to be the result of TGF-β-induced activation of the SNAIL transcription factor, a key mediator of EMT, and repression of epithelial markers, such as E-cadherin [12]. The Smad protein family is known to play a key role in the TGF-β signaling, particularly Smad4, the ubiquitination of which is a key regulatory step in TGF-β signaling [13]. Indeed, loss of inactivation of Smad4 has been linked with multiple cancers, including pancreatic, colorectal, and gastrointestinal cancers [14-16].

Protein ubiquitination is a reversible, post-translational modification that regulates various aspects of cellular physiology, including protein degradation and cell signaling [17]. Deubiquitinating enzymes (DUBs) are ubiquitin-specific proteases that can cleave ubiquitin from its substrate [18]. Among approximately 100 DUBs encoded by the human genome, the ubiquitin-specific peptidase 9, X-linked (USP9X/FAM), is implicated in multiple physiological pathways [19]. USP9X has been shown to regulate multiple cellular functions, and increased expression of USP9X in tumors is significantly associated with poor prognosis for patients with multiple myeloma [20]. Numerous targets of USP9X have been identified so far, including AF-6, β -catenin, NUAK1, MARK4, ErbB2, EFA6, Smad4, Mcl1, ASK1 and survivin [21].

Recently, microRNA (miRNA) mimics and anti-sense microRNAs have been focused on as potential therapeutics for HCC due to their stability and predominant uptake by the liver [22]. MiRNAs bind to the 3′ untranslated region (UTR) to suppress translation of target genes [23]. An increasing body of evidence indicates that miR-26b is downregulated in breast cancer [24], nasopharyngeal carcinoma [25], colorectal cancer [26] and in hepatocellular carcinoma [27]. miR-26b has also been associated with hepatocellular carcinoma development and worst outcome after liver cancer therapy [28,29]. However, to date, the role of miR-26b in hepatocellular carcinoma tumorigenesis and metastasis is incompletely understood.

The goal of the current study was to investigate the role of miR-26b in HCC. We found that the expression of miR-26b was decreased in HCC cells and tissues from HCC patients with advanced grades of disease. Mimicry of miR-26b in Huh7 and Hep3B cells resulted in diminished proliferation, migration and invasiveness, accompanied by a low expression level of USP9X. Further, we identified USP9X as a target of miR-26b, which was confirmed by luciferase analysis, Western blotting examination. Our study provides evidence that miR-26b acts as tumor suppressor in HCC, and is an important negative regulator of USP9X.

## Methods

### Human HCC tissue

Liver samples collected from patients with HCC and control liver samples were collected for research purposes, with the patient’s prior consent and approval from the Second Affiliated Hospital of Guangzhou Medical University and the Guangzhou Women and Children’s Medical Center.

### Cell culture

The following human HCC cell lines were studied: HepG2, MHCC97H, Hep3B, MHCC97L, HCCC9810, BEL-7402, Huh7 and QGY-7703. All cells were grown in DMEM medium (Invitrogen, Carlsbad, CA, USA) supplemented with 10% fetal bovine serum (HyClone, Logan, Utah, USA) and 1% penicillin/streptomycin.

### Manipulation of miR-26b expression levels

The miR-26b mimics, negative control (micr*ON*™ miRNA Mimic Negative Controls and micr*OFF*™ miRNA Inhibitor Negtive Controls) and miR-26b inhibitor were purchased from RiboBio (Guangzhou, Guangdong, China). The final concentrations of transfection is 20 nM.

### Construction of USP9X plasmids

The region of the human USP9X 3′-UTR, from 1041 to 1486 were generated by PCR amplification from DNA isolated from HepG2 cells. The amplified fragment was then cloned into the pEGFP-C1 (Clontech, Mountain View, CA, USA) and pGL3 vector (Promega, Madison, Wisconsin, USA). The primers selected were as follows: USP9X -3′UTR-GFP-up, 5′ CCGCTCGAGCCAGTGACGTGGAAGTCATC 3′; USP9X -3′UTR-GFP-dn, 5′ CGGGGTACCCACCACAGGACAAAAAGTTCTTC 3′; USP9X -3′UTR-luc-up, 5′ CGGGGTACCCCAGTGACGTGGAAGTCATC 3′; and USP9X -3′UTR-luc-dn, 5′ CCGCTCGAGCACCACAGGACAAAAAGTTCTTC 3′.

### Cell transfections

Transfection of the plasmids, miRNA and miRNA inhibitor was performed using Lipofectamine 2000 (Invitrogen, Carlsbad, USA) according to the manufacturer’s instructions.

### Western blotting

Cells were harvested in sampling buffer (62.5 mmol/L Tris-HCl [pH 6.8], 10% glycerol, 2% SDS) and heated for 5 min at 100°C. The concentration of extracted proteins was determined by the Bradford assay using a commercial kit (Bio-Rad, Berkeley, CA, USA ). Equal quantities of protein were separated by electrophoresis on 12% SDS/polyacrylamide gels and transferred onto polyvinylidene difluoride membranes (Roche, Indianapolis, Indiana, USA). The membranes were then probed with rabbit primary antibodies against USP9X(#5751S), E-cadherin(#9835S), Vimentin(#3877P) and GFP(#2955S) (Cell Signaling Technology, Danvers, Massachusetts, USA). The expression level of the target protein was determined with horseradish peroxidase-conjugated anti-rabbit/anti-mouse IgG (#31212/ #31160) and enhanced chemiluminescence (Pierce, Rockford, Illinois, USA), according to the manufacturer’s protocol. The membranes were stripped and reprobed with an anti-β-actin mouse monoclonal antibody (BOSTER, Wuhan, Hubei, China) to serve as a loading control.

### RNA extraction and real-time quantitative PCR

Total miRNA from cultured cells and fresh surgical HCC tissues was extracted using the mirVana miRNA Isolation Kit (# AM1561, Ambion, Austin, Texas, USA), according to the manufacturer’s protocol. Complimentary DNA was synthesized from 5 ng of total RNA using the TaqMan miRNA reverse transcription kit (Applied Biosystems, Foster City, California, USA). The expression level of miR-26b was quantified using a miRNA-specific TaqMan MiRNA Assay Kit (Applied Biosystems), using an Applied Biosystems 7500 Sequence Detection system. The expression level of miRNA was defined based on the threshold cycle (Ct), and relative expression levels were calculated using the 2^-ΔΔCt^ method, using the expression level of the U6 small nuclear RNA as a reference gene.

### Cell migration assay

The migration assay was performed using a transwell chamber, consisting of 8 mm membrane filter inserts (Corning, New York, USA) coated with Matrigel (BD Biosciences, California, USA). Briefly, cells were trypsinized and suspended in serum-free medium. Next, 1.5 × 10^5^ cells were added to the upper chamber, and the lower chamber was filled with medium containing 10% FBS. After 36 h incubation, cells that had invaded the lower chamber were fixed with 4% paraformaldehyde, stained with hematoxylin, and counted using a microscope.

### Wound healing assay

The wound healing assay was performed using HepG2 and Huh7 cells. Cells were trypsinized and seeded in equal numbers into 6-well tissue culture plates, and allowed to grow until confluent (approximately 24 h). Following serum starvation for 24 h, an artificial homogenous wound (‘scratch’) was created onto the cell monolayer with a sterile 100 μL tip. After scratching, the cells were washed with serum-free medium, complete media was added, and microscopic images (20× magnification) of the cells were collected at 0, 12, and 24 h.

### 3D morphogenesis assay

Twenty-four-well dishes were coated with Growth Factor Reduced Matrigel (BD Biosciences, California, USA), and covered with growth medium supplemented with 2% Matrigel. Cells were trypsinized and seeded at a density of 10^4^ cells/well. The medium was replaced with 2% Matrigel every 3 to 4 days and microscopic images (20× magnification) were captured at 2 day intervals for 2 to 3 weeks.

### Dual luciferase reporter assay

Cells (3.5 × 10^4^) were seeded in triplicate in 24-well plates and allowed to settle over 24 h. Next, 100 ng of pGL3-USP9X-3′UTR (wt/mut), or control-luciferase plasmid plus 1 ng of pRL-TK renilla plasmid (# E2810. Promega, Madison, Wisconsin, USA) were transfected into the cells using Lipofectamine 2000 (Invitrogen Co., Carlsbad, California, USA), according to the manufacturer’s recommendations. Luciferase and renilla signals were measured 48 h after transfection using the Dual Luciferase Reporter Assay Kit (Promega, Madison, Wisconsin, USA), according to the manufacturer’s protocol. Three independent experiments were performed and the data are presented as the mean ± SD.

### Statistical analysis

A two-tailed Student’s t-test was used to evaluate the statistical significance of the differences between two groups of data in all pertinent experiments. A P-value less than 0.05 was considered to be statistically significant. All statistical analyses were performed using the SPSS 13.0 (IBM) statistical software package.

### Microscopy

The microscopy we have used as:

Zeiss Axio Imager A1——Zeiss, Oberkochen, Germany

Zeiss Axiovert 40c——Zeiss, Oberkochen, Germany

Axiovert 200——Zeis, Oberkochen, Germany

## Results

### MiR-26b downregulation correlates with progression of human hepatocellular carcinoma

**Figure 1:**
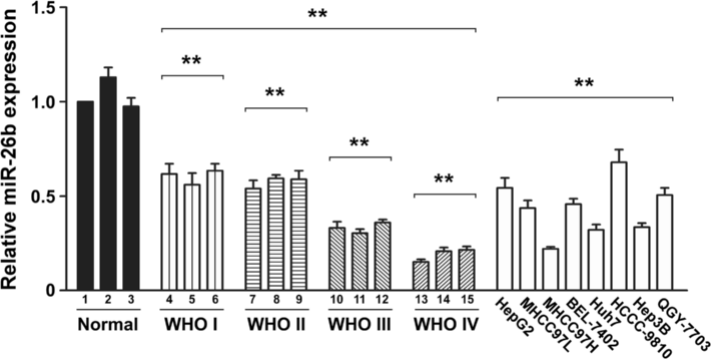
**Downregulation of miR-26b in human HCC cell lines and tissues.** The relative expression level of miR-26b was examined in normal human liver tissue (Normal), and HCC tissues at different WHO classifications. The expression level of miR-26b was also compared in eight HCC cell lines: HepG2, MHCC97L, MHCC97H, Bel-7402, Huh7, HCCC-9810, Hep3B, and QGY-7703. All experiments were repeated at least three times. Each bar represents the mean of three independent experiments. * *P* < 0.05. The average expression level of miR-26b were compared between the normal tissues and the HCC tissues, the normal tissues and HCC cell lines.

An increasing body of evidence indicates miR-26b is downregulated in several cancers, including hepatocellular carcinoma. But its role in the tumorigenesis of HCC remains poorly understood. In our early studies, the result of real-time PCR analysis showed that the expression level of miR-26b was markedly downregulated in all eight of the HCC cell lines analyzed (HepG2, MHCC97L MHCC97H, BEL-7402, Huh7, HCCC-9810, Hep3B and QGY-7703), in comparison with the expression levels in normal liver tissue from three patients( *P* < 0.001) (Figure 1). The clinical relevance of the downregulated miR-26b was studied in twelve archived clinical HCC samples, classified as grade I to IV according to the WHO definitions. Our data show that miR-26b levels downregulated in HCC tissues, compared with the normal tissues( *P* < 0.001). Moreover, the expression of miR-26b is negatively correlates with the severity and progression of HCC. As shown in Figure 1, we can see that the miR-26b expression still remained high in grade I and II tumors, but became markedly lower in grade III and IV tumors. Taken together these results indicate that miR-26b may be an tumor inhibitor in the progression of human hepatocellular carcinoma.

### MiR-26b suppresses the invasiveness of HCC cells

**Figure 2:**
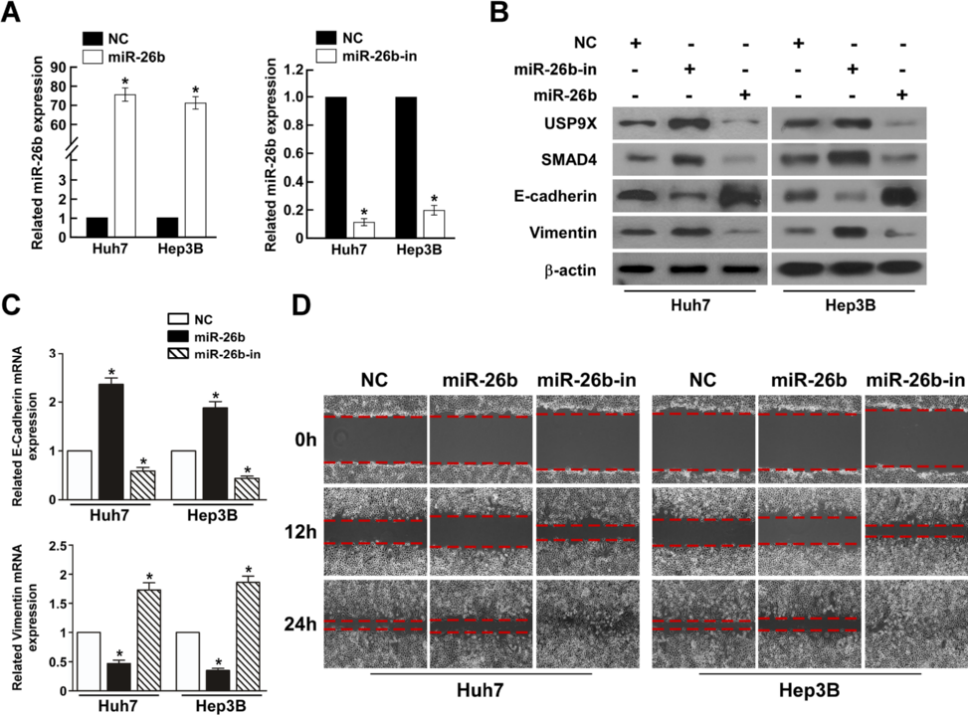
**Increased levels of miR-26b suppress mobolity and E-cadherin expression in HCC cell lines. (A)** Real-time PCR analysis of expression of miR-26b in indicated cells. U6 was used as a loading control. Error bars represent mean ± SD from three independent experiments. **(B)** Western blotting analysis of USP9X, SMAD4, E-cadherin and vimentin expression in indicated cells. **(C)** Real-time PCR analysis of expression of E-cadherin and vimentin in indicated cells. GAPDH was used as a loading control. Error bars represent mean ± SD from three independent experiments. **(D)** A wound closure assay was used to assess the mobility of Huh7 and Hep3B cells treated with the empty vector, miR-26b mimic or miR-26 inhibition (original magnification: ×20;).

To investigate the biological function of miR-26b in HCC cells, we used Huh7/Hep3B cell line with medium-level of miR-26b expression for both knockdown and overexpression modifications, by which subsequent functional analyses could be performed comparatively among miR-26b-overexpressing, -silenced, and NC control cells that had the same parental genetic background. We transfected the HCC cell lines Huh 7 and Hep3B with miR-26b mimic oligonucleotides to increase the endogenous level of miR-26b, or miR-26b complementary oligonucleotides to decrease the levels of miR-26b (Figures 2A). The first parameter we studied was the expression of genes related to cell invasion. Western blotting and Real-time PCR analyses showed that the epithelial marker E-cadherin was upregulated following transfection with the miR-26b mimic, and decreased with oligonucleotides against miR-26b (Figures 2B and C). On the other hand, that mesenchymal marker vimentin was downregulated following transfection with the miR-26b mimic, and was increased by oligonucleotides against miR-26b (Figures 2B and C).Next, we assessed the effect of manipulating cellular miR-26b levels on the migration of Huh 7 and Hep3B cells using wound healing and cell migration assays. The wound healing assay demonstrated that ectopic expression of miR-26b slowed down the migration of Huh 7 and Hep3B cells, while inhibiting endogenous miR-26b dramatically accelerated migration (Figure 2D).

**Figure 3:**
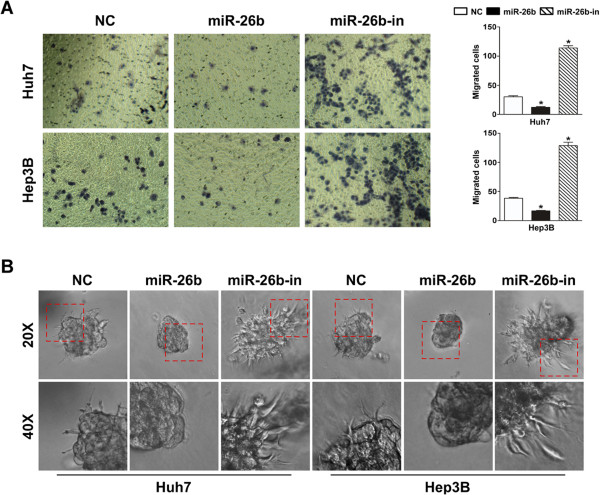
**Increased levels of miR-26b suppress EMT in HCC cell lines. (A)** The invasive properties of HCC cells treated with the empty vector, miR-26b mimic and miR-26b inhibitor were analyzed using a cell invasion assay in transwell chambers. Representatives images of cells that had migrated into the lower chamber are shown (left panel; original magnification: ×400), and quantitative data are also presented (right panel). The average number of cells per field of view from three different experiments are plotted. **(B)** The invasive properties of HCC cells treated with empty vector, miR-26b mimic or miR-26b inhibitor were also performed using a 3D spheroid invasion assay. Representative images of cells after 8 days in culture are shown (upper; original magnification:: ×200, bottom; original magnification:: ×400).

The cell migration assay showed that overexpression of miR-26b reduced the number of migratory cells, while inhibition of miR-26b increased the number of migratory HCC cells (Figure 3A). Strikingly, in a 3D spheroid invasion assay, decreased levels of miR-26b in HCC cells were associated with morphologies typical of highly aggressive and invasive cells, presenting more outward projections compared with the control cells. Conversely, elevated levels of miR-26b caused the cells to be immotile and to display spheroid morphologies (Figure 3B). What’ s more, an E-cadherin staining showed the corresponding result (Additional file 1: Figure S1).

### MiR-26b directly targets USP9X in HCC cells

**Figure 4:**
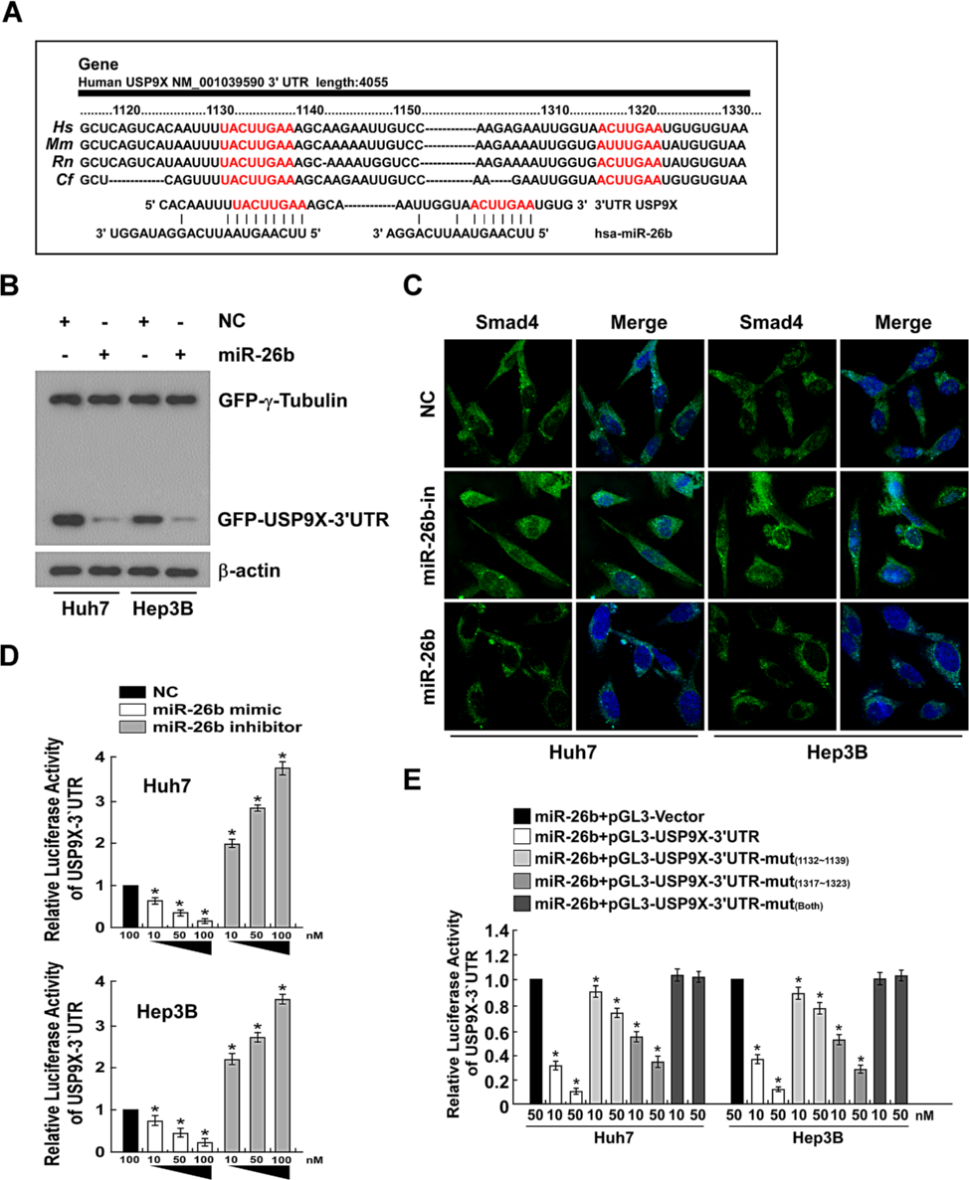
**miR-26b downregulates USP9X expression via directly targeting the USP9X 3′UTR. (A)** Sequence alignment of the 3′UTR in Hs(human), Mm(mouse), Rn(rat), and Cf (dog) with miR-26b indicates that miR-26b targets USP9X expression. **(B)** Representative western blots of GFP expression in Huh7 and Hep3B cells under the indicated experimental conditions. **(C)** Representative fluorescent microscope images of Smad4 expression in Huh7 and Hep3B cells following treatment with the empty vector, miR-26b inhibitor and miR-26b mimic are shown. **(D)** Luciferase activity assay of indicated cells transfected with the pGL3-USP9X-3′UTR reporter with increasing amounts (10, 50 ,100 nM) of miR-26b mimic- or miR-26b inhibitor-oligonucleotides. **(E)** Luciferase assays on Huh7 or Hep3B HCC cells transfected with the pGL3 control reporter, pGL3-USP9X-3′UTR reporter, or pGL3-USP9X-3′UTR-mu reporters and increasing amounts of miR-26b mimic oligonucleotides (10, 50 nM), as indicated. Error bars represent mean 6 SD from three independent experiments. *P < 0.05.

It is known that Smad4 ubiquitination is a key regulatory step in the TGF-β signaling pathway, and it was later shown that the DUB USP9X was required for Smad activity [30]. In mammalian cells and Xenopus embryos, USP9X sustains both TGF-β and BMP signaling by deubiquitinating Smad4 and by counteracting the inhibitory activity of Ecto/Tif1-c [31]. Researches have identified FAM (especially USP9x), a deubiquitinase acting as essential and evolutionarily conserved component in TGF-β signal pathway [32]. We analysis with the use of two publicly available algorithms (TargetScan and miRanda) revealed that USP9X is a theoretical target gene of miR-26b (Figure 4A). Subsequent Western blotting and immunofluorescence staining results showed that miR-26b mimicry dramatically decreased the expression level of USP9X, and inhibition of miR-26b increased USP9X protein expression in HCC cells (Figure 2B and Figure 4B). But the result of Real-time PCR showed that the mRNA levels of USP9X remains unchanged after miR-26b manipulation( data not showed). While the effect of USP9X on ubiquitination of Smad4 after miR-26b modulation as shown in Additional file 2: Figure S2.

To determine whether the miR-26b-induced downregulation of USP9X was mediated through the 3′UTR containing the miR-26b binding site, we subcloned the USP9X 3′UTR fragment into PEGFP-C1 and pGL3 dual luciferase reporter vectors. As our Western blotting analysis showed, elevating the endogenous levels of miR-26b only decreased expression of the GFP vector containing the USP9X with 3′UTR, but had no effect on GFP-r-tubulin expression (Figure 4B), suggesting that miR-26b specifically affected the 3′UTR of USP9X. Furthermore, western blotting and immunofluorescence staining results showed that the abundance of Smad4 was significantly increased when miR-26b was suppressed, and was decreased in the presence of elevated miR-26b expression (Figure 2B and 4C). Moreover, a consistent and dose-dependent reduction in luciferase activity was observed following miR-26b transfection in both Huh7 and Hep3B cells, whereas the repressive effect of miR-26b on the luciferase activity of USP9X 3′UTR was abolished by miR-26b inhibition (Figure 4D), and point mutations in the tentative miR-26b-binding seed region in USP9X 3′-UTR abrogated the suppressive effect of USP9X mediated by miR-26b (Figure 4E). Last but not the least, real-time PCR result showed that the expression level of SNAIL mRNA was significantly decreased in either miR-26b orUSP9X siRNA over-expressed HCC cells (Additional file 3: Figure S3). Collectively, these results demonstrate that miR-26b directly acts through the 3′UTR of USP9X in HCC cells.

### USP9X plays an important role in miR-26b suppressed invasiveness of HCC cells

**Figure 5:**
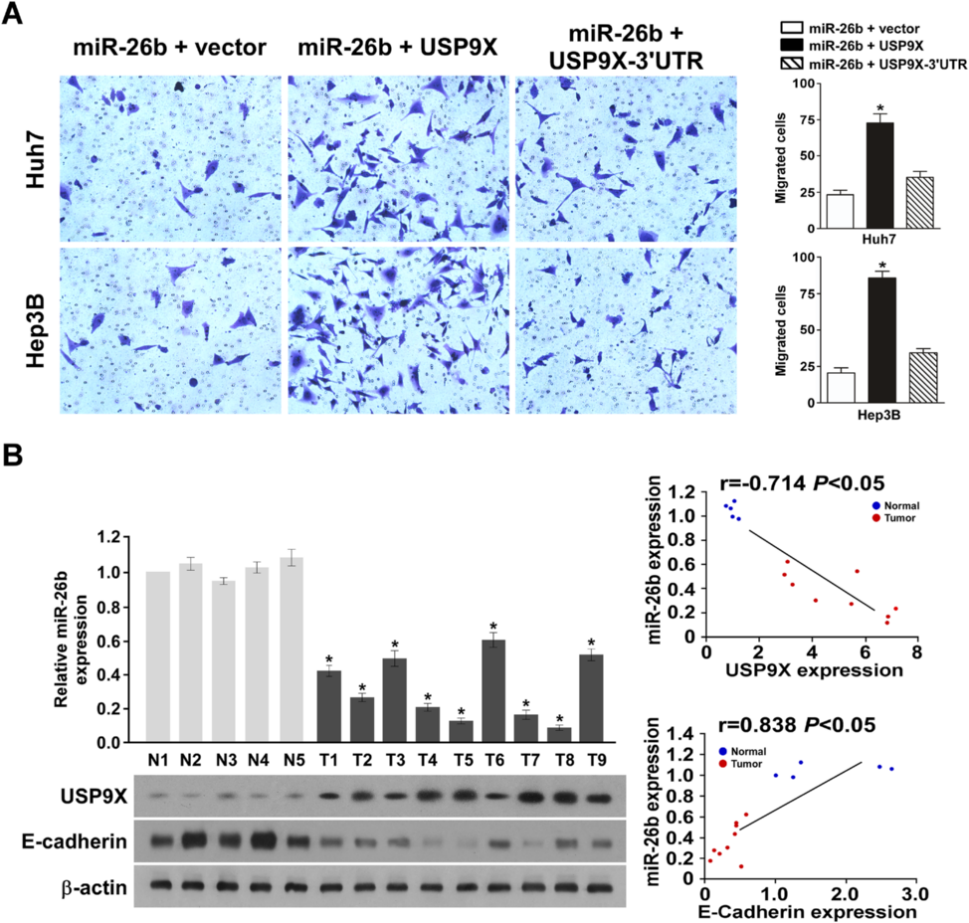
**miR-26b downregulates USP9X via directly targeting the USP9X 3′UTR. (A)** Representative images of cells in the lower section of a transwell chamber are shown to demonstrate the invasive properties of Huh7 and Hep3B cells when transfected with a mixed miR-26b and USP9X vector, or a mixed miR-26b and USP9X-3′UTR (left panel). Quantitative analyses of migrated cells are also shown ( right panel); data are plotted as the average number of cells per field of view from three different experiments (original magnification: ×400). **(B)** The relative expression level of miR-26b is shown for five normal liver tissue samples (N), and nine HCC liver tissue samples (upper right panel). Below, the corresponding expression levels of USP9X, E-cadherin and β-actin are shown in the same samples. Linear regression analyses of miR-26b expression with USP9X and E-cadherin are also presented (right panel).

To further investigate the role of increased USP9X expression in cells with suppressed miR-26b levels and increased invasiveness, the effects of USP9X without 3′UTR and USP9X with 3′UTR were examined in cells transfected with miR-26b mimics. The migration assay showed that co-transfection of miR-26b and USP9X without 3′UTR significantly improved the migration rate of both Huh7 and Hep3B cells. However, the combination of USP9X with 3′UTR and miR-26b had no obvious effect on migration, compared to cells transfected with miR-26b and vector (Figure 5A). Furthermore, cells transfected with miR-26b-in and USP9X siRNA significantly decreased the migration rate when compared to cells transfected with miR-26b-in and scramble in Huh7 (Additional file 4: Figure S4). Furthermore, we re-introduced the USP9X siRNA into the miR-26b inhibbited HCC cancer cells. As shown in Additional file 5: Figure S5, ectopic expression of USP9X siRNA increased the expression levels of E-cadherin and decreased the expression levels of vimentin.

In order to confirm the link between miR-26b, USP9X and EMT in HCC, we studied the expression level of miR-26b, USP9X and E-cadherin in primary liver tumor tissue from patients with HCC. For this analysis, five freshly prepared normal human liver samples were compared with nine samples from patients with HCC. As shown in Figure 5B, miR-26b expression and E-cadherin protein expression were markedly downregulated in HCC tissue, but the expression level of USP9X was higher, compared with normal human liver tissue. Furthermore, linear regression analysis showed that miR-26b expression positively correlated with E-cadherin expression (r = 0.838, *P* < 0.05), and negatively correlated with USP9X (r = –0.714, *P* < 0.05).

## Discussion

The goal of the current study was to investigate the role of miR-26b in EMT and the metastasis of HCC. During EMT, polarized epithelial cells acquire a mesenchymal phenotype; in the context of cancer, this transformation is associated with tumor invasiveness, metastasis, and resistance to chemotherapy [5,7,23,24]. A number of cytokines and growth factors are known to be induced during EMT, which amplifies the EMT program and promotes cell migration. These factors include the cytokines TGF-β, Wnt, Notch ligands, interleukin-like EMT-inducers [6], hepatocyte growth factor [8], epidermal growth factor [30] and platelet-derived growth factor [31].

The levels of transcription factors driving EMT are controlled by miRNAs [33]. While miR-26a and miR-26b have been reported can regulate the NF-κB, TGF-β signal pathways [34]. And miR-26b was downregulated in hepatic tumors, as compared to paired noncancerous tissue [35]. Similarly, patients whose tumors had low miR-26 expression had a shorter survival time, although they were more likely to respond to interferon-α than patients whose tumors had high miR-26 expression [28]. Several studies have confirmed that miR-26a and miR-26b are downregulated in NPC (nasopharyngeal carcinoma) [36], CRC (colorectal cancer) [37]. Mechanistically, miR-26 is thought to be growth suppressive; it has been shown that miR-26b acts through c-myc to block the G1/S transition [19].

The role of TGFβ in EMT, tumor invasiveness and metastasis has been firmly established by in vitro and in vivo studies [8,32]. Interestingly, TGF-β-induced activation of Smad complexes (particularly Smad3 and 4) have been shown to play a crucial role during the induction of EMT [32-35]. Ubiquitination of Smad4 is known to be a key regulatory step in the TGF-β signaling pathway, leading to the identification of the DUB USP9X as a required factor for Smad activity [30,32,37]. In mammalian cells and Xenopus embryos, USP9X sustains both TGF-β and BMP signaling by deubiquitinating Smad4 and counteracting the inhibitory activity of Ecto/Tif1-c [32]. Here, we identified USP9X as a novel target of miR-26b, such that miR-26b interacted with the 3′-UTR of USP9X to suppress its expression, with resultant effects on Smad4 expression and the TGF-β signaling pathway.

There is usually an inverse relationship between the expression level of a particular miRNA and its target [31]^.^ Our conclusion that USP9X is a novel target gene of miR-26b is supported by several pieces of evidence: (1) the 3′-UTR of both human and murine USP9X mRNA contains a putative miR-26b binding site; (2) miR-26b suppresses the activity of a luciferase reporter gene fused with the 3′-UTR of USP9X mRNA, which is dependent on the miR-26b binding sequence; (3) miR-26b represses the endogenous expression of human/murine USP9X at both the mRNA and protein level. Our follow-up studies indicated that the ability of miR-26b to inhibit cancer cell invasion was mediated through its ability to downregulate USP9X expression.

USP9X was previously reported to regulate Smad4 transcriptional activity positively, thus we hypothesized that decreased USP9X expression would attenuate Smad4 function and TGF-β responsiveness in HCC cell lines [38]. Our data demonstrate that transfection with a miR-26b mimic decreased Smad4 expression in both Huh7 and Hep3B cells, while transfection of a miR-26b inhibitor upregulated Smad4 expression. These results suggest that the effect of miR-26b on HCC cells may be mediated through Smad4, with a resultant effect on the TGF-β signaling pathway.

## Conclusion

In conclusion, we have identified miR-26b as a tumor suppressive miRNA in human HCC, which acts at least in part through the repression of USP9X. Mechanistically, our data suggests a model in which miR-26b binds to the 3′-UTR of USP9X to inhibit its expression. Decreased USP9X expression leads to an attenuation of Smad4 function and TGF-β responsiveness, inhibiting the EMT and growth of HCC cells. Our data suggests that miR-26b may play some roles in the EMT of HCC cells, although future studies are required to confirm this.

## Competing interests

The authors declare that they have no competing interests.

## Authors’ contributions

GS and YL carried out the molecular genetic studies, participated in the sequence alignment and drafted the manuscript. XY carried out the cell proliferation assays. JZ participated in immunoassays. ZX participated in the real-time PCR and immunoassays. HJ design of the study and performed the statistical analysis and helped to draft the manuscript. All authors read and approved the final manuscript.

## Pre-publication history

The pre-publication history for this paper can be accessed here:

http://www.biomedcentral.com/1471-2407/14/393/prepub

## Supplementary Material

Additional file 1: Figure S1 Inhibiting miR-26b decrease the expression of E-cadherin Representative fluorescent microscope images of E-cadherin expression in Huh7 and cells following treatment with the empty vector, miR-26b inhibitor and miR-26b mimic are shown. Click here for file

Additional file 2: Figure S2 The influence of miR-26b on Smad4 ubiquitnation. Ectopic expression of miR-26b increased the ubiquitination levels of Smad4( Ub-Smad4 is a 75-KDa band reactive to the anti-Smad4 antibody). Click here for file

Additional file 3: Figure S3 Relative SNAIL mRNA expression Real-time PCR analysis of expression of SNAIL in either miR-26b overexpression or USP9X siliencing in indicated cells. Click here for file

Additional file 4: Figure S4 Siliencing the USP9X in Huh7 cells has the similar effect as miR-26b overexpression. Representative images of cells in the lower section of a transwell chamber are shown to demonstrate the invasive properties of Huh7 when Knocking-down USP9X. Click here for file

Additional file 5: Figure S5 Silencing USP9X inducing the same phenocopy the effects of miR-26b overexpression on HCC cells. Ectopic expression of USP9X siRNA increased the expression levels of E-cadherin and decreased the expression levels of vimentin. Click here for file
