# Supplementary figures and images for: MicroRNA-26b inhibits epithelial-mesenchymal transition in hepatocellular carcinoma by targeting USP9X
Source: BMC Cancer. 2014 Jun 2;14:393. doi: 10.1186/1471-2407-14-393 (PMC4062892; doi:10.1186/1471-2407-14-393)

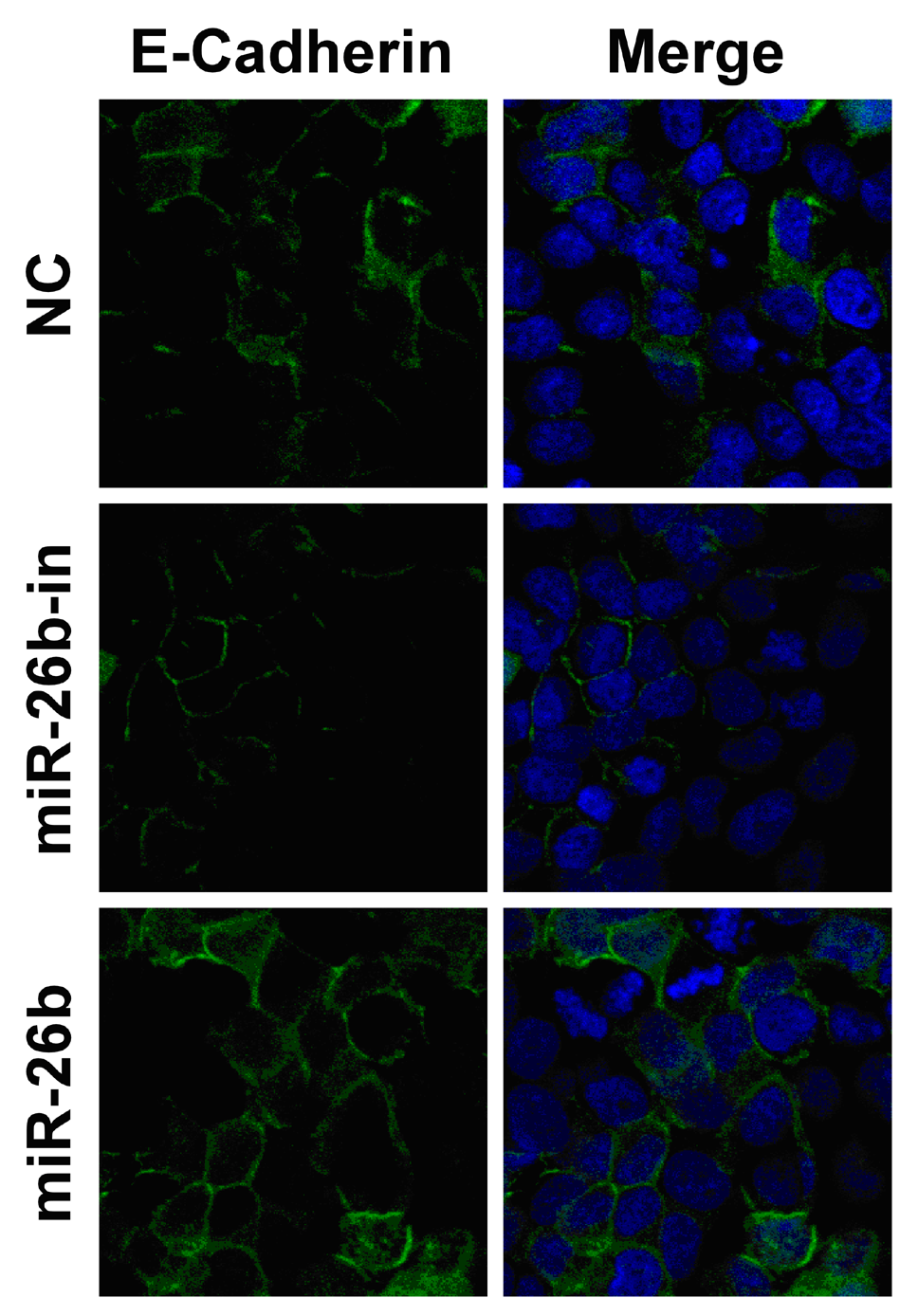

Supplement: Additional file 1: Figure S1 — Inhibiting miR-26b decrease the expression of E-cadherin Representative fluorescent microscope images of E-cadherin expression in Huh7 and cells following treatment with the empty vector, miR-26b inhibitor and miR-26b mimic are shown. [file 1471-2407-14-393-S1.tiff]

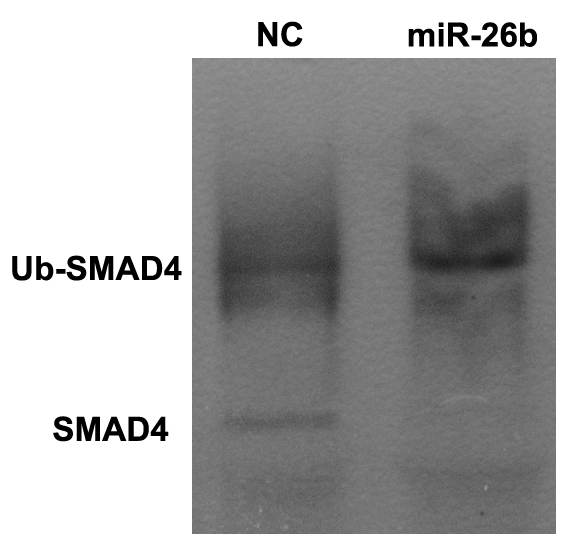

Supplement: Additional file 2: Figure S2 — The influence of miR-26b on Smad4 ubiquitnation. Ectopic expression of miR-26b increased the ubiquitination levels of Smad4( Ub-Smad4 is a 75-KDa band reactive to the anti-Smad4 antibody). [file 1471-2407-14-393-S2.tiff]

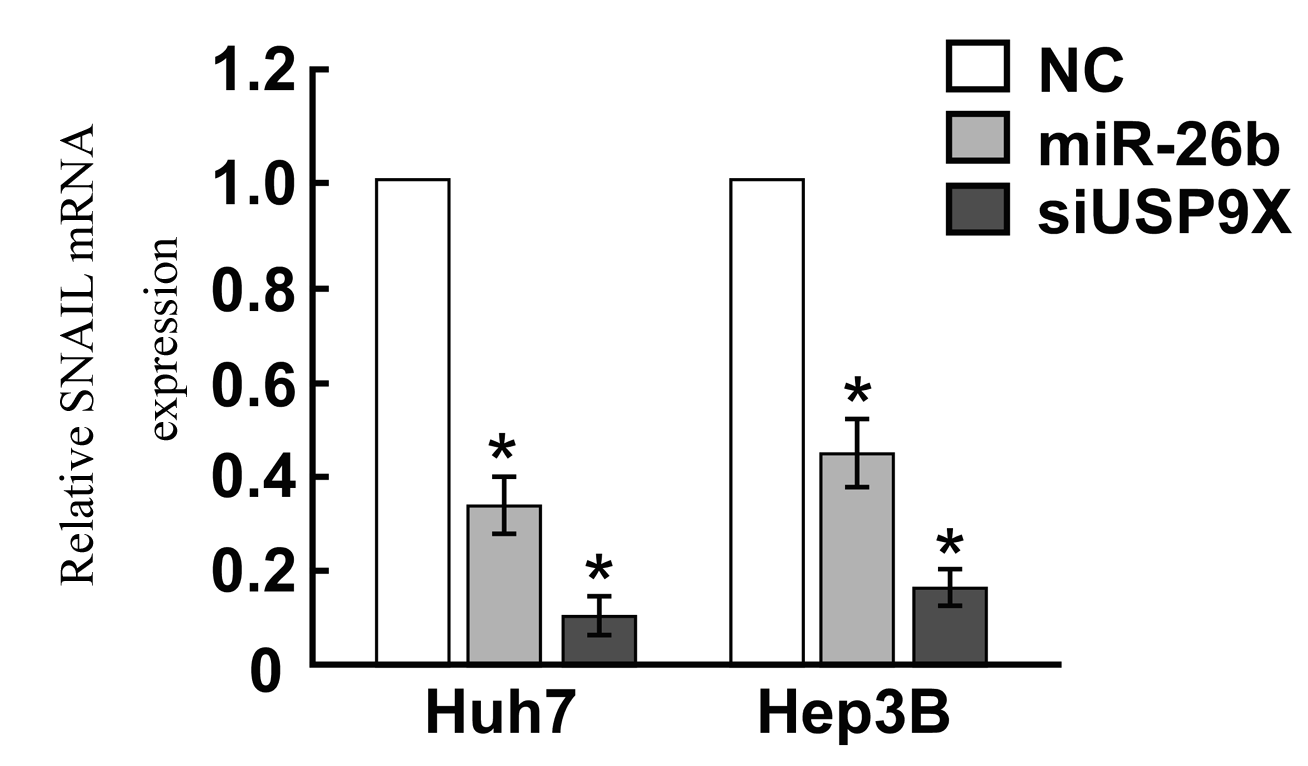

Supplement: Additional file 3: Figure S3 — Relative SNAIL mRNA expression Real-time PCR analysis of expression of SNAIL in either miR-26b overexpression or USP9X siliencing in indicated cells. [file 1471-2407-14-393-S3.tiff]

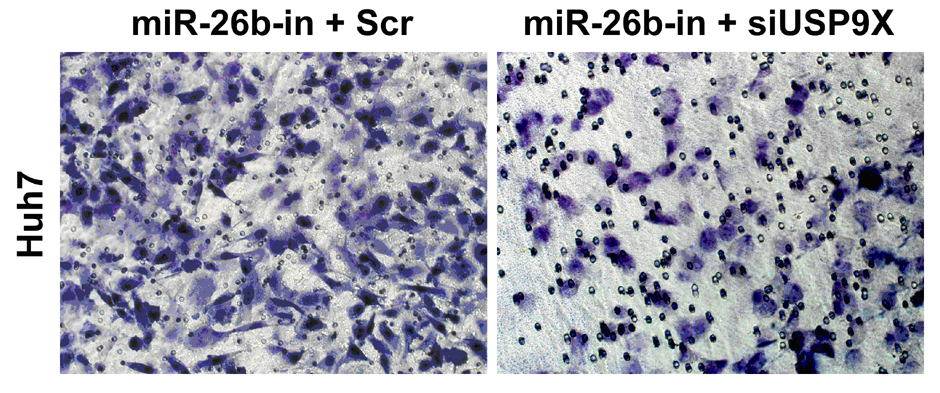

Supplement: Additional file 4: Figure S4 — Siliencing the USP9X in Huh7 cells has the similar effect as miR-26b overexpression. Representative images of cells in the lower section of a transwell chamber are shown to demonstrate the invasive properties of Huh7 when Knocking-down USP9X. [file 1471-2407-14-393-S4.tiff]

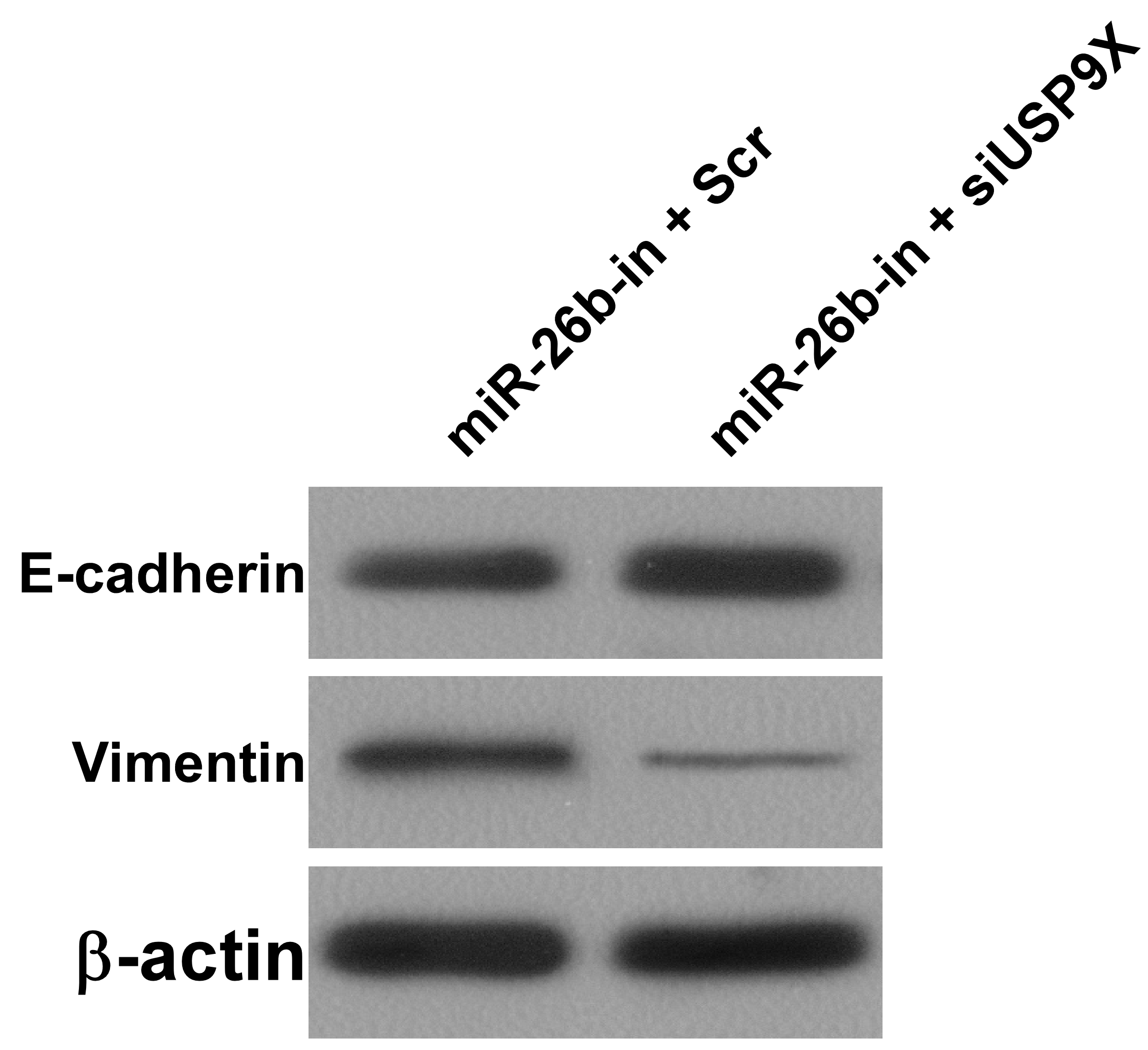

Supplement: Additional file 5: Figure S5 — Silencing USP9X inducing the same phenocopy the effects of miR-26b overexpression on HCC cells. Ectopic expression of USP9X siRNA increased the expression levels of E-cadherin and decreased the expression levels of vimentin. [file 1471-2407-14-393-S5.tiff]
